# Influence of Levamisole and Other Angiogenesis Inhibitors on Angiogenesis and Endothelial Cell Morphology *in Vitro*

**DOI:** 10.3390/cancers5030762

**Published:** 2013-06-24

**Authors:** Tina Friis, Anne-Marie Engel, Christine D. Bendiksen, Line S. Larsen, Gunnar Houen

**Affiliations:** Department of Clinical Biochemistry, Immunology and Genetics, Statens Serum Institut, Artillerivej 5, DK-2300 Copenhagen, Denmark

**Keywords:** angiogenesis, inhibitor, endothelial cell, morphology, *in vitro*, proliferation, signaling pathway, levamisole

## Abstract

Angiogenesis, the formation of new blood vessels from existing vessels is required for many physiological processes and for growth of solid tumors. Initiated by hypoxia, angiogenesis involves binding of angiogenic factors to endothelial cell (EC) receptors and activation of cellular signaling, differentiation, migration, proliferation, interconnection and canalization of ECs, remodeling of the extracellular matrix and stabilization of newly formed vessels. Experimentally, these processes can be studied by several *in vitro* and *in vivo* assays focusing on different steps in the process. *In vitro*, ECs form networks of capillary-like tubes when propagated for three days in coculture with fibroblasts. The tube formation is dependent on vascular endothelial growth factor (VEGF) and omission of VEGF from the culture medium results in the formation of clusters of undifferentiated ECs. Addition of angiogenesis inhibitors to the coculture system disrupts endothelial network formation and influences EC morphology in two distinct ways. Treatment with antibodies to VEGF, soluble VEGF receptor, the VEGF receptor tyrosine kinase inhibitor SU5614, protein tyrosine phosphatase inhibitor (PTPI) IV or levamisole results in the formation of EC clusters of variable size. This cluster morphology is a result of inhibited EC differentiation and levamisole can be inferred to influence and block VEGF signaling. Treatment with platelet factor 4, thrombospondin, rapamycin, suramin, TNP-470, salubrinal, PTPI I, PTPI II, clodronate, NSC87877 or non-steriodal anti-inflammatory drugs (NSAIDs) results in the formation of short cords of ECs, which suggests that these inhibitors have an influence on later steps in the angiogenic process, such as EC proliferation and migration. A humanized antibody to VEGF is one of a few angiogenesis inhibitors used clinically for treatment of cancer. Levamisole is approved for clinical treatment of cancer and is interesting with respect to anti-angiogenic activity *in vivo* since it inhibits ECs *in vitro* with a morphology resembling that obtained with antibodies to VEGF.

## 1. Introduction

In this review we describe basic aspects of angiogenesis and models for studying angiogenesis with an emphasis on *in vitro* coculture assays with endothelial cells and fibroblasts.

### 1.1. Angiogenesis

Angiogenesis, the development of new blood capillaries and vessels, is required in physiological processes such as embryogenesis, tissue repair (wound healing) and the female reproductive cycle, but is also involved in pathological conditions such as psoriasis, rheumatoid arthritis, diabetic retinopathy and tumor formation [1,2,3,4].

**Figure 1:**
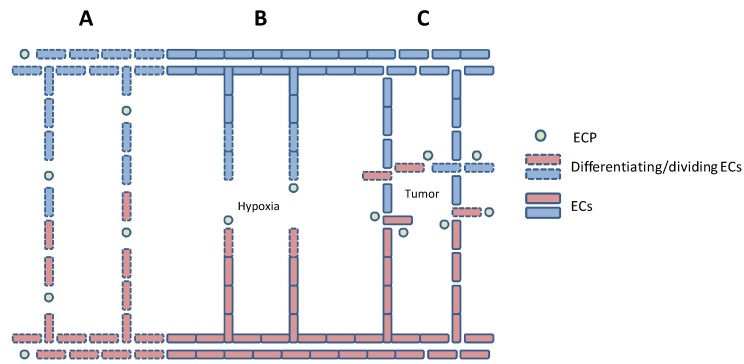
Schematic presentation of vasculogenesis (**A**), physiological angiogenesis (**B**) and tumor angiogenesis (**C**).

During early embryonic development, the vascular system is established from mesodermal endothelial precursor cells (EPCs) by vasculogenesis [5]. This early vasculature expands and forms more complex networks by angiogenesis (Figure 1) involving multiple simultaneous processes including: increased vessel permeability and activation of proteases that degrade the basement membrane and extracellular matrix (ECM), binding of growth factors to their receptors on endothelial cells (ECs), differentiation and elongation of ECs, EC migration and proliferation towards the angiogenesis-stimulating source, EC lumen formation and stabilisation of newly formed vessels. Although angiogenesis mainly occurs by “sprouting” from existing vessels, it may also involve splitting (intussusception) and bridging of vessels [6].

A major parameter regulating angiogenesis is the tissue O_2_ concentration. However, the supply of nutrients and the disposal of waste products such as CO_2_ are also important in the regulation of angiogenesis. The physiological outcome is the developmental growth of tissues in fetal life and maintenance of tissue homeostasis after birth. These processes are coordinated by an array of extracellular growth factors and signalling molecules acting in an autocrine and paracrine fashion, and by intracellular signalling molecules controlling the actions of transcription factors, translation factors and metabolic pathways [1,2,3,4,5,6].

**Figure 2:**
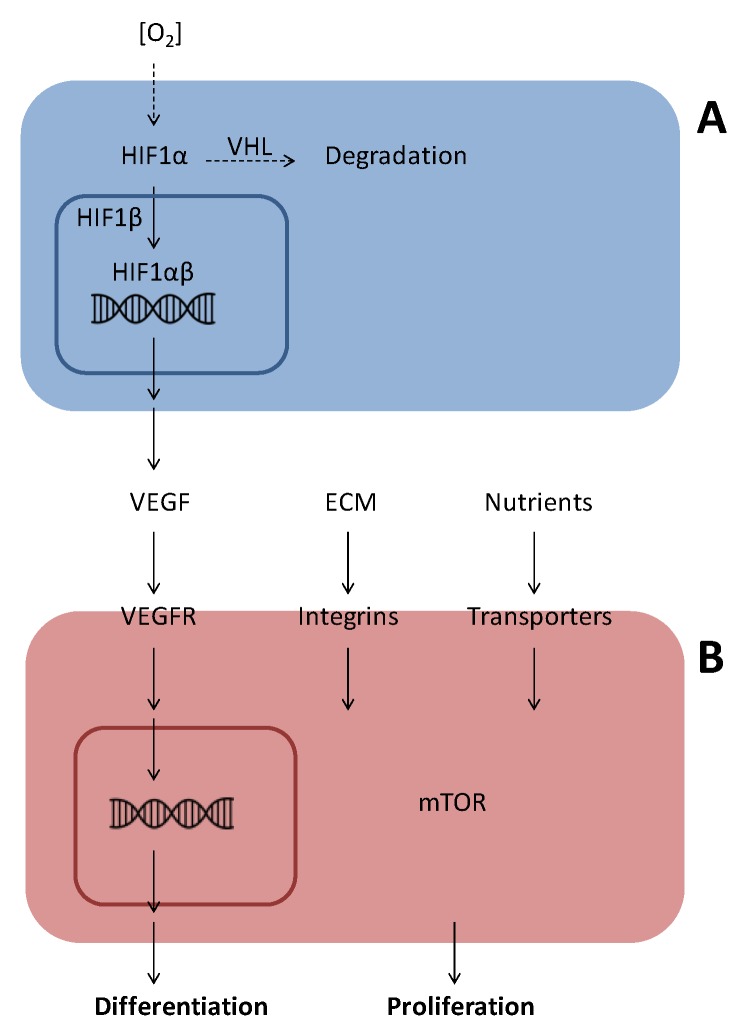
Oxygen sensing by HIF (**A**) and signal transduction by VEGF (**B**). (**A**) Under conditions of low oxygen concentrations in the cytoplasm, HIF1α undergoes nuclear translocation and associates with HIF1β in the nucleus, where the dimeric HIF1 stimulates transcription of genes with hypoxia-responsive elements (HRE) in their promoters. Under normoxia, HIF1α is hydroxylated on specific prolines and this leads to association with the VHL protein, an E3 ligase, which stimulates ubiquitin-dependent proteasomal degradation of HIF1α. (**B**) Binding of VEGF to its plasma membrane receptor (VEGFR) initiates a pleiotrophic response with autophosphorylation, activation of associated adaptor proteins and phosphorylation of membrane-associated signal transducing proteins. These signalling pathways lead to nuclear translocation of transcription factors and activation of gene transcription resulting in cellular protein synthesis, differentiation and/or proliferation.

The transcription factor hypoxia-inducible factor 1 (HIF1) is a central player in O_2_ sensing and regulation of angiogenesis [7,8]. HIF1 is a heterodimer composed of a β subunit and one of three α subunits. Under normoxic conditions, HIF1α associates with the von Hippel Lindau tumor suppressor (VHL) and is degraded via the ubiquitin proteasome pathway. HIF1α-VHL association is regulated by proline hydroxylation and lysine acetylation in the oxygen-dependent degradation (ODD) domain of HIF1α. During hypoxia HIF1α is translocated to the nucleus, where it interacts with HIF1β and several coactivators to induce the transcription of genes important for cell survival and angiogenesis, including vascular endothelial growth factor (VEGF) (Figure 2) [7,8].

### 1.2. Angiogenic Factors

Angiogenesis is controlled by a balance between pro-angiogenic and anti-angiogenic factors in the local environment [1,2,3,4,5,6]. Angiogenesis-stimulating factors can be divided in directly and indirectly acting factors. The directly acting angiogenic factors include VEGF [9,10] and angiopoietins (Angs) [11], which act mainly on ECs, and interleukin-8 (IL-8), which acts on other cell types as well [12]. The indirectly acting angiogenic factors include fibroblast growth factors (FGFs) [13,14] and tumor necrosis factor alfa (TNFα) [15] and stimulate different types of non-ECs (e.g., fibroblasts, monocytes, macrophages, neutrophils or tumor cells) to produce directly acting angiogenic factors. Many pro-angiogenic factors are involved in the complex regulation of angiogenesis [1,2,3,4,16,17], but here we will focus on VEGF and Angs.

#### 1.2.1. Vascular Endothelial Growth Factor (VEGF)

The most important angiogenic factor is VEGF and angiogenesis is initiated by binding of VEGF to receptors present on ECs (Figure 2) [9,10]. The human VEGF family consists of 5 dimeric glycoproteins with heparin binding sites: VEGF (also called VEGF-A), VEGF-B, VEGF-C, VEGF-D and placenta growth factor (PlGF). In addition, alternative splicing of the VEGF-A gene can generate different isoforms composed of 121, 145, 165, 189 and 206 amino acids, of which VEGF_165_ is the dominant isoform involved in natural and pathologic angiogenesis.

The members of the VEGF family bind in distinct ways to three cell surface receptor tyrosine kinases (VEGFR1-3) [9,10]. VEGFR1 is present on ECs, monocytes and trophoblast cells, VEGFR2 is found on ECs and VEGFR3 is present on lymphatic ECs, vascular ECs engaged in active angiogenesis, macrophages, osteoblasts and neuronal progenitors. ECs also express neuropilins, which act as coreceptors and differentially promote interaction of VEGF isoforms with their receptors [16].

Upon ligand-induced activation, the VEGF receptors undergo homodimerisation and auto- and trans-phosphorylation reactions in distinct ways. Binding of VEGF to VEGFR2 initiates a number of intracellular signal transduction pathways, which induce EC differentiation, migration, proliferation and increased vessel permeability (Figure 2). VEGFR1 binds VEGF with higher affinity than VEGFR2, but is not or only weakly autophosphorylated upon ligand binding and acts as a decoy receptor.

Regulation of VEGFR expression depends on several factors, notably O_2_ concentration and other parameters, which regulate HIF1 [10].

#### 1.2.2. Angiopoietins (Angs)

Ang 1–4 constitute another family of directly acting angiogenic factors, which are regulated by hypoxia [11]. Their receptors, Tie1 and Tie2, have different ligand binding properties and are tyrosine kinases, which undergo dimerization and phosphorylation reactions in response to ligand binding. This activates many of the same cellular signalling pathways, which are activated by VEGF. Ang1 is produced by perivascular cells and tumor cells in several splice variants, which act differentially on ECs through Tie2 to induce endothelial survival and vessel stabilisation. Ang2 is produced by ECs and acts as an autocrine antagonist and modulator of Ang1. Ang3 and Ang4 are mouse/human orthologs, which bind and activate their receptors in a manner similar to Ang1. Their role in angiogenesis is less well characterised.

## 2. Endothelial Cell Differentiation and Migration

Activation of VEGFR signalling induces an increase in vascular permeability and secretion of proteases, which degrade the surrounding basement membrane and ECM [1,2,3,4]. This allows immigration from the blood stream of endothelial progenitor cells (EPCs), which differentiate under the influence of VEGF (Figure 1). Activation of VEGFR2 and association with integrin αVβ3 stimulate EC migration and proliferation [10]. This VEGFR2 integrin association is important for focal adhesion, cellular signalling and recruitment of the actin-binding vinculin, involved in initiation of EC migration. During migration, ECs adhere to fibrinogen, fibronectin, laminin and vitronectin in the ECM via heterodimeric integrins. After migration and proliferation, vacuoles in the ECs coalesce to form a lumen and adjacent cells adhere through formation of tight junctions [17].

## 3. Vessel Stabilization

Nascent vessels mature into capillaries by recruitment of pericyte precursors and into arteries or veins by recruitment of smooth muscle precursors. The pericytes and smooth muscle cells (mural cells) stabilize the newly formed vessels and participate in the regulation of blood flow through them [1,2,3,4]. Several factors, including Ang1, platelet-derived growth factor β (PDGFβ) and transforming growth factor β (TGFβ), are important for the assembly of nascent vessels and the production of ECM around them [1,2,3,4].

## 4. Tumor-Induced Angiogenesis

Under pathological circumstances, such as tumor growth, the ECM is degraded and the balance is switched towards a predominance of pro-angiogenic factors [1,2,3,4]. Initially, many tumors grow around existing blood vessels, where their demands for oxygen and nutrients can be met by simple diffusion. At this stage, tumor cells do not secrete angiogenesis-stimulating factors, but they may induce EC production of Ang2, which, in the absence of VEGF, promotes vessel destabilisation and regression.

When a tumor has grown to a size of about 1–2 mm in diameter, the centre of the tumor becomes hypoxic, as its demand for oxygen is no longer fulfilled by simple diffusion. Consequently, HIF1α and HIF1β associate and initiate the transcription of genes coding for VEGF, FGF, IL-8, PDGF and Ang2, which together push the angiogenic switch in favour of angiogenesis [1,2,3,4,7,8].

Tumor-induced angiogenesis is an unbalanced process, which results in excessive formation of new blood vessels [1,2,3,4]. Some are termed mosaic vessels, because the innermost layer of the vessel wall is composed not only of ECs, but also of tumor cells. The abnormal tumor vessels are convoluted, have variable diameters and express surface markers in a non-uniform manner. Moreover, they are leaky and without orientation, and consequently the blood flow delivered by such vessels will be chaotic and irregular (Figure 1).

## 5. *In vitro* Models of Angiogenesis

*In vitro* models are important for studying basic aspects of angiogenesis and for reproducible screening of novel potential angiogenesis inhibitors. However, most *in vitro* angiogenesis assays do not cover all steps of the angiogenic process.

### 5.1. Proliferation Assays

Simple ways of detecting EC proliferation include counting in a microscope, counting by flourescence-activated cell sorting (FACS), measurements of DNA synthesis and measurements of metabolic activity (e.g., dye reduction) [18,19,20,21]. In this type of monoculture assays the ECs grow as not fully differentiated cells, but they allow easy screening and evaluation of the effects of many compounds on EC growth.

### 5.2. Migration Assays

EC migration can be investigated in various assays, where ECs are seeded on surfaces and allowed to migrate and proliferate [22,23,24]. EC migration can also be measured using a Boyden chamber, composed of an upper and a lower chamber separated by a membrane permeable for migrating cells. ECs, seeded in the upper chamber, migrate across the membrane in response to attractants added to the lower chamber and are quantified by simple counting or other techniques [25,26,27]. Compared with simple proliferation assays, migration assays add another dimension and allow easy screening of many compounds. Wound healing assays, where confluent monolayers of ECs are mechanically or chemically wounded in a standardised manner, combine proliferation and migration and also allow easy screening end evaluation of the effects of many compounds [28].

### 5.3. Three-Dimensional Gel Assays

ECs form tubular structures when propagated in a sandwich between two fibrin gels and when seeded in a three-dimensional gel containing collagen I and laminin or the frequently used Matrigel (ECM components and growth factors produced by the mouse Engelbreth-Holm-Swarm Sarcoma) [29,30,31,32]. A minor disadvantage of these assays is that Matrigel is a heterogenous mixture and newer assays employ three-dimensional networks with defined components [33,34].

### 5.4. Coculture Assays with ECs and Fibroblasts

In 1999, Bishop *et al.* [35] introduced a new *in vitro* angiogenesis assay, in which ECs formed capillary-like tubular structures upon coculturing with fibroblasts for 14 days in 24-well plates. This coculture assay covers EC proliferation and tube formation and the length and width of immuno-stained endothelial tubes are measured by image analysis of randomly selected areas.

We have further developed this coculture system into a quantitative and qualitative ELISA-based angiogenesis assay [36]. In this assay, normal human dermal fibroblasts (NHDF) are cultured for three days in 96 wells plates. Then, human umbilical vein ECs (HUVECs) are seeded on top of the confluent fibroblast layer and propagated in coculture for three days in a growth factor-enriched medium, TFSM2. The cells are fixed with ice cold ethanol and non-specific binding is blocked by incubation with Tris-buffer containing Tween 20 and sodium chloride. The total amount of cells is estimated by using a monoclonal antibody to double-stranded DNA and ECs are detected with monoclonal antibodies to CD31 (PECAM-1), von Willebrand Factor (vWF) and collagen IV (Col IV). Alkaline phosphatase-conjugated secondary antibodies and *p*-nitrophenyl phosphate (*p*NPP) in alkaline substrate buffer are used for quantification. The absorbance is read at 405 nm with background subtraction at 650 nm. Bound antibodies are subsequently visualized by development with 5-bromo-4-chloro-3-indolyl phosphate/nitro blue tetrazolium (BCIP/NBT) after aspiration of the *p*NPP solution. This combination allows microscopic inspection of EC morphology in the same wells that are used for ELISA.

Electron microscopy has shown that the ECs propagated in coculture with fibroblasts form capillary-like tubes with lumen after three days of cocultivation and that the number of tubes with lumen increases when cells are propagated in coculture for ten days [37]. The ECs in the capillary-like tubes retain important ultrastructural and physiological properties of normal ECs, in that they are connected via tight junctions and contain transport vesicles and Weibel-Palade bodies, the storage compartments for vWF [37].

**Figure 3:**
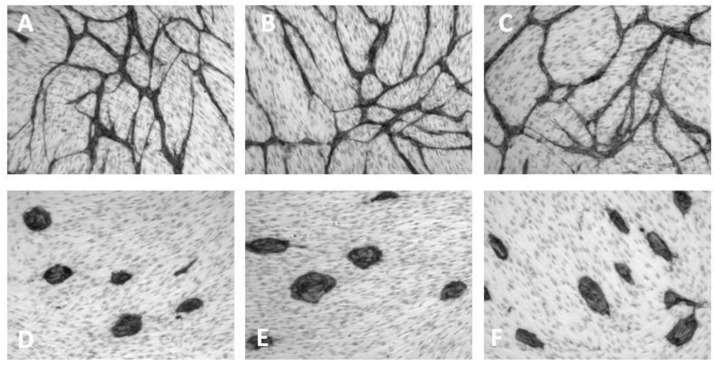
VEGF-dependent capillary-like tube formation of HUVECs propagated for three days in coculture with fibroblasts in (**A**) TFSM2 medium, (**B**) TFSM2 medium without bFGF, (**C**) TFSM2 medium + 20 µg/mL goat anti-bFGF, (**D**) TFSM2 medium without bFGF and VEGF, (**E**) TFSM2 medium without VEGF, (**F**) TFSM2 medium + 5 µg/mL goat anti-VEGF. Cell nuclei are counterstained with Mayer’s acid haematoxylin.

**Figure 4:**
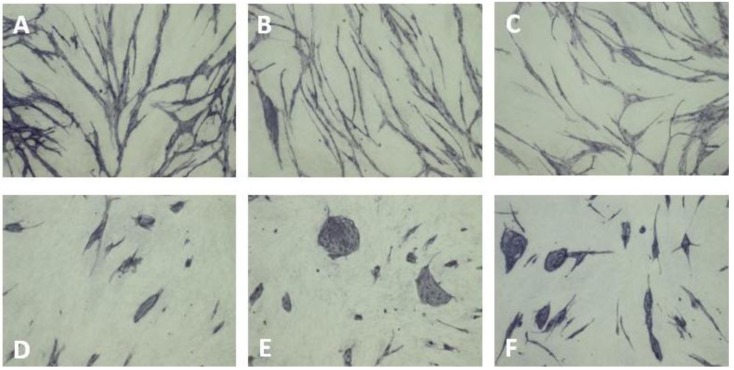
VEGF-dependent capillary-like tube formation of HMVECs propagated for three days in coculture with fibroblasts in (**A**) HMVEC5 medium, (**B**) HMVEC5 medium without bFGF, (**C**) HMVEC5 medium + 20 µg/mL goat anti-bFGF, (**D**) HMVEC5 medium without bFGF and VEGF, (**E**) HMVEC5 medium without VEGF, (**F**) HMVEC5 medium + 40 µg/mL goat anti-VEGF.

In this assay, an elaborate network of differentiated ECs is formed when HUVECs are grown on a layer of living NHDFs in the presence of VEGF (Figure 3A). However, in the absence of VEGF, clusters of non-differentiated ECs are formed (Figure 3D–E). The same morphological features are observed when replacing HUVECs with human microvascular ECs (HMVECs) in a coculture system, where NHDFs and HMVECs are cocultured for 4 days in HMVEC5 medium (an EGF-depleted TFSM2 medium with 5% fetal calf serum (FCS) and 100 ng/mL VEGF instead of 2% FCS and 10 ng/mL VEGF as used for HUVECs (Figure 4A,D–E). Recently, Sarkanen *et al.* improved the sensitivity of this assay by increasing the coculture period from three to six days [38] and other variations have been described which allow real time microscopy of ECs [39].

Interestingly, when the ECs are seeded on top of ethanol-fixed NHDFs, they do not form networks of differentiated cells, even in the presence of VEGF [36]. This indicates that interaction with other cell types, either directly or through excreted substances, is essential for differentiation into capillary-forming cells.

Removal of bFGF from the culture medium has no effect on the growth of ECs in the coculture assay (Figure 3B, Figure 4B). This is not because excess bFGF is being excreted from the NHDF as no change is seen upon addition of antibodies to bFGF (Figure 3C, Figure 4C). This observation can be explained by the NHDFs being confluent (and having an adequate O_2_ supply) and agrees with bFGF being an indirectly acting angiogenic factor, which induces VEGF secretion by fibroblasts. Some studies show a minor degree of EC differentiation/proliferation in the absence of exogenously added VEGF [39]. Presumably, a basal VEGF excretion is present under many culture conditions and may vary from assay to assay.

Addition of well-known angiogenesis inhibitors to the coculture angiogenesis assay was found to specifically inhibit the ECs without affecting the fibroblasts as described below [36,37].

## 6. Inhibition of Angiogenesis

Inhibition of angiogenesis is an appealing approach for cancer treatment, since solid tumors are dependent on an adequate blood supply in order to grow and metastazise [1]. The risk of development of cellular resistance to angiogenesis inhibitors can be considered to be low, as the target for the anti-angiogenic therapy is not the genetically unstable tumor cells but non-transformed ECs involved in the formation of new blood vessels. However, tumor cells are relatively resistant to hypoxia due to a shift to anaerobic metabolism [40]. Moreover, tumor cells may also activate epithelial-mesenchymal transition (EMT), recruit bone-marrow derived stem cells, and induce cooption of existing vasculature [41]. Despite this, angiogenesis inhibitors have been found to be valuable as a supplement to existing cancer treatment.

Angiogenesis can be inhibited by affecting one or more steps in the angiogenic process. A number of angiogenesis inhibitors with different inhibitory mechanisms have been identified and can be divided in two groups: Endogenous inhibitors found in the human body and exogenous inhibitors, which comprise antibodies and low molecular weight compounds (Table 1). Below, selected angiogenesis inhibitors are reviewed and their effects in the coculture assay described.

**Table 1:** Selected angiogenesis inhibitors discussed in this review.

Endogenous angiogenesis inhibitors	Exogenous angiogenesis inhibitors
Angiostatin	Fumagillin/TNP-470
Antithrombin	NSAIDs
CXCL4	PTPIs *
Endostatin	Rapamycin
Thrombospondin	SU5614
Vasostatin	Suramin
sVEGFR1	VEGF antibodies

* Protein tyrosine phosphatase inhibitors.

## 7. Endogenous Inhibitors of Angiogenesis

Endogenous inhibitors of angiogenesis include various components of the VEGF signalling system, other growth factors and ECM components (Table 1). These may act directly or indirectly.

### 7.1. Angiostatin

Angiostatin, a 38 kDa fragment of plasminogen, containing the first four kringle domains, has been shown to be an inhibitor of endothelial proliferation and migration *in vitro* as well as tumor growth and metastasis *in vivo* [42,43].

We have not observed an inhibitory effect of angiostatin (15–20 µg/mL) on proliferation of HUVECs in monoculture or on tube formation of HUVECs in coculture with NHDFs. This indicates that angiostatin *in vivo* may act as an indirect inhibitor of angiogenesis, exerting its effect through other cell types than ECs.

### 7.2. Antithrombin

Cleaved and latent forms of antithrombin have been reported to inhibit VEGF-induced EC proliferation *in vitro* and tumor growth *in vivo* [44].

Treatment with 50–100 µg/mL cleaved or native antithrombin purified from human plasma had no effect on EC tube formation in the coculture assay, indicating that antithrombin is an indirect inhibitor of angiogenesis.

### 7.3. Endostatin and Other Globular Collagen Domains

A number of collagen *C*-terminal globular non-collagenous domains have been reported to show anti-angiogenic anti-tumor activity *in vivo* [45,46,47]. Arresten, canstatin and tumstatin are *C*-terminal fragments of the α_1_, α_2_, and α_3_ chains of collagen IV with molecular weights of 26 kDa, 24 kDa and 30 kDa, respectively [45]. Restin is a 22 kDa *C*-terminal fragment of the α_1_ chain of collagen XV [46] and endostatin is a 20 kDa *C*-terminal fragment of the α_1_ chain of collagen XVIII [47].

Endostatin in a concentration of 15–20 µg/mL had no effect in the coculture assay. This indicates that endostatin is an indirect angiogenesis inhibitor but could also be due to the use of macrovascular HUVECs, since Taddei *et al.* found that endostatin inhibited the proliferation of microvascular but not macrovascular ECs [48].

### 7.4. Platelet Factor 4

Platelet factor 4 (PF4), a CXC chemokine (CXCL4), and its isoform CXCL4L1 are potent inhibitors of angiogenesis [49]. They act directly through the receptor CXCR3 on ECs, but also appear to be capable of interaction with proteoglycans [49,50,51]. CXCL4L1, differing from CXCL4 by three amino acid residues in the *C*-terminal end, appears to be the more potent form [49] and their angiostatic activity can be mimicked by a *C*-terminal fragment [49,52].

**Figure 5:**
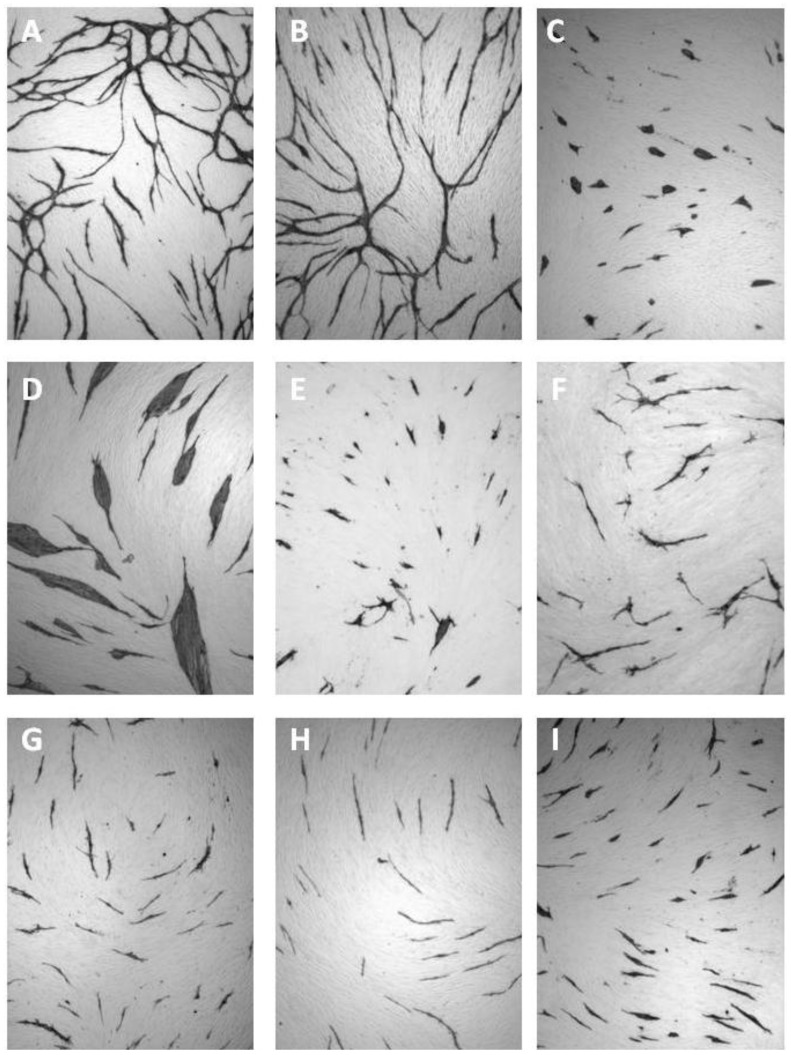
Effect of angiogenesis inhibitors in the coculture assay. HUVECs propagated in coculture with fibroblasts in TFSM2 medium (**A**) or TFSM2 medium with: (**B**) PBS, (**C**) 10 µM SU5614, (**D**) 1 mM levamisole, (**E**) 5 µg/mL PF4, (**F**) 1 µM TNP470, (**G**) 2.5 mM indomethacin, (**H**) 0.1 µg/mL rapamycin, or (**I**) 0.1 mM suramin.

In the coculture assay we found that 5 µg/mL PF4 inhibited EC proliferation as seen by the formation of small isolated cords of cells (Figure 5E).

### 7.5. Thrombospondin

Thrombospondins (TSPs) are multifunctional ECM proteins, which have been found to regulate EC proliferation and to induce apoptosis in ECs [53,54]. Their mechanisms of actions are complex and involve binding to integrins, integrin-associated protein (CD47) and CD36 [53,54,55].

In agreement with these observations, preliminary experiments show that 12 µg/mL TSP induced the formation of short cords of ECs in the coculture assay.

### 7.6. Vasostatin

Calreticulin and an *N*-terminal fragment of it, vasostatin, have been reported to inhibit EC proliferation *in vitro* as well as angiogenesis and tumor growth *in vivo* [56,57,58]. The mechanism of action remains uncertain, but may involve interaction with laminin [59].

We have not been able to demonstrate an anti-angiogenic effect of calreticulin (16 µg/mL) in the coculture assay, indicating that it may act as an indirect inhibitor of angiogenesis *in vivo*.

### 7.7. VEGF Isoforms and Decoy Receptors

Alternatively spliced isoforms of VEGF exist, which may activate VEGFRs differently or not at all and thus act as modulators or inhibitors of angiogenesis [9,10]. VEGFR1 and VEGFR2 also exist in alternatively spliced, soluble forms which prevent binding of VEGFA to VEGFR2 and VEGFC to VEGFR3, thus inhibiting angiogenesis and lymphangiogenesis, respectively [9,10]. In the coculture assay we have demonstrated that a soluble truncated form of VEGFR1, purified from human placenta, inhibits EC differentiation and tube formation.

## 8. Exogenous Inhibitors of Angiogenesis

Angiogenesis inhibitors of exogenous origin are a heterogenous group and include both directly and indirectly acting compounds (Table 1).

### 8.1. Fumagillin and TNP-470

The angiogenesis-inhibiting activity of fumagillin was discovered as a consequence of Aspergillus fumigatus contamination in an EC culture [60]. The synthetic analogue *O*-(chloroacetylcarbamoyl)fumagillol (TNP470) and a water soluble *N*-(2-hydroxypropyl)methacrylamide-conjugated form of TNP470 (caplostatin) were found to be more potent and less toxic than fumagillin [61,62,63,64]. The anti-angiogenic mechanism of fumagillin and TNP470 is unclear, but they have been shown to covalently bind and inactivate methionine aminopeptidase 2 (MetAP2) [65,66] and to inhibit EC proliferation, migration and tube formation in a number of *in vitro* and *in vivo* assays of angiogenesis [61,62,63,64,67,68,69]. In our coculture assay, 1 µM TNP470 inhibited EC proliferation resulting in cells with short cord morphology [37] (Figure 5F).

### 8.2. Non-Steroidal Anti-Inflammatory Drugs (NSAIDs)

NSAIDs have been demonstrated to inhibit EC proliferation, migration and tube formation *in vitro* as well as tumor growth and angiogenesis *in vivo*, presumably by inhibition of cyclooxygenases, for which they have different inhibitory selectivity [70,71,72].

In the coculture assay, 2.5 mM of the NSAIDs aspirin, ibuprofen and indomethacin inhibited EC proliferation and EC interconnection, resulting in short cord morphology (Figure 5G).

### 8.3. Levamisole and Other Phosphatase Inhibitors

Levamisole is an antihelminthic agent but has also been used for adjuvant chemotherapy of colorectal cancer [73,74,75]. We have shown that levamisole inhibits the proliferation of ECs in monoculture, and the differentiation and capillary network formation of ECs in coculture with fibroblasts, yielding clusters of non-differentiated cells [76] (Figure 5D). This anti-angiogenic property of levamisole was confirmed by Sarkanen *et al.* using a similar assay [38].

Furthermore, we synthesized various *N*-alkylated levamisole analogues and tested these in the coculture assay together with commercially available derivatives. The majority of the *N*-alkylated levamisole derivatives disrupted endothelial network formation less efficiently than levamisole and gave rise to a mixed morphology of clusters and cords, but *N*-methyllevamisole and *p-*bromolevamisole were both more effective inhibitors than levamisole [77].

The anti-angiogenic mechanism of levamisole in not known, but may involve the VEGF signalling pathway, since it induces an endothelial cluster morphology in the coculture assay resembling cocultures without VEGF or cocultures treated with VEGF antibodies (Figure 3, Figure 4, Figure 5). Levamisole has been reported to induce apoptosis in cultured ECs [78] and myeloma cell lines [79] and a derivative (2-benzyl-6-(4'-fluorophenyl)-5-thiocyanato-imidazo[2,1-b][1],[3],[4]thiadiazole) induced apoptosis in leukemia cell lines [80]. A screening for cellular targets of various chemicals identified Bcl-associated death promoter (BAD), Bcl-xL, p21, mitogen-activated protein kinase 2 (MAPK2) and LIM domain kinase 2 (Limk2) as possible targets for levamisole [81]. Since levamisole is known to be an inhibitor of alkaline phosphatase, we investigated the possible relation between phosphatase inhibition and anti-angiogenic activity. We did not find a correlation between inhibition of alkaline phosphatases and angiostatic activity for levamisole and the *N*-alkylated derivatives [77]. However, we found that several phosphatase inhibitors showed considerable angiogenesis inhibition *in vitro* in the coculture assay [82]. Salubrinal, NSC87877, clodronate, PTP inhibitor I and II induced the ECs to form short cords, indicating impairment of EC proliferation and elongation. PTP inhibitor IV inhibited EC differentiation and induced cluster formation, while ibandronate resulted in a mixed morphology. PTP inhibitor IV has been reported to inhibit angiogenesis by targeting SHP-2 [83].

### 8.4. Rapamycin

Rapamycin is an immunosuppressive agent that has been demonstrated to inhibit EC proliferation and migration *in vitro* and to inhibit growth factor- and tumor-induced angiogenesis in different *in vivo* assays [84,85,86]. These effects are mediated by blocking signalling through mTOR (mammalian target of rapamycin), a general sensor of cellular nutrient and energy status [87,88] (Figure 2).

In the coculture assay 0.1–10 µg/mL rapamycin inhibited EC proliferation resulting in a morphology of short cords (Figure 5H).

### 8.5. SU5614 and Other Kinase Inhibitors

Many protein kinase inhibitors have been developed for inhibition of angiogenesis and/or cell growth [1,4,9,89,90]. SU5614, a tyrosine kinase inhibitor, which can block the VEGFR-associated tyrosine kinase activity, has been demonstrated to inhibit EC tube formation *in vitro* in a long-term coculture assay of ECs and fibroblasts [90,91,92].

In our coculture assay 10 µM SU5614 resulted in clusters of low differentiated ECs similar to cells grown in the absence of VEGF [37] (Figure 5C).

### 8.6. Suramin

Suramin is a polysulfonated naphthylurea originally developed as an anti-trypanosomal agent [93]. Later, suramin was reported to inhibit EC proliferation and migration *in vitro* as well as growth factor- and tumor-induced angiogenesis in a number of *in vivo* angiogenesis assays [94,95,96]. Suramin has been demonstrated to influence angiogenesis directly by binding to VEGF and indirectly by binding bFGF and PDGF [94,95,96].

In the coculture assay 0.1 mM Suramin was an effective inhibitor of proliferation yielding isolated ECs with the short cord morphology [37] (Figure 5I).

### 8.7. VEGF Antibodies

Neutralizing antibodies to VEGF or soluble receptor constructs are efficient angiogenesis inhibitors [1,3,4,9]. In the HUVEC and NHDF coculture assay and the HMVEC and NHDF coculture assay, 5 µg/mL and 40 µg/mL goat antibodies to human VEGF (Figure 3F, Figure 4F) inhibited EC differentiation resulting in EC clusters with a morphology identical to that seen in cocultures propagated without VEGF [37] (Figure 3D–E and Figure 4D–E).

## 9. *In vitro* Assays of Angiogenesis

*In vitro* assays can only simulate some aspects of angiogenesis and *in vivo* assays are used to study angiogenesis under more physiological conditions. Such assays are also mandatory for preclinical testing of potential angiogenesis inhibitors. A number of *in vivo* assays have been developed including the chorioallantoic membrane (CAM) assay, the cornea assay, the hindlimb ischemia model, matrigel-based implantation assays and different animal models of tumor-angiogenesis [97,98,99,100,101].

### 9.1. The Chicken CAM Assay

A commonly used model for *in vivo* angiogenesis is the chicken chorioallantoic membrane (CAM) assay [97,98]. In order to engraft an angiogenic agent in the CAM, a window is either cut in the shell of the fertilised egg or the embryo is transferred to a petri dish. This allows easy access to continuous visual determination of neoangiogenesis in the CAM. The degree of neovascularization can be evaluated by a visual scoring system or by image analysis. The assay is suitable for continuous non-invasive screening and it allows the use of xenografts as the CAM lacks a potent immune system. However, discrimination between remodelled pre-existing vessels and vessels formed by neo-angiogenesis may be difficult. Non-specific inflammatory responses, vascular remodelling or CAM wounding during the assay time might give rise to false positive results.

### 9.2. Cornea Assays

The cornea is avascular by nature and the cornea assay therefore allows the detection of neoangiogenesis without interference of pre-existing vasculature [97,98]. In the cornea of rabbits, mice or rats small pockets are made by surgery, and a growth factor-containing pellet or a tumor biopsy is implanted in the pocket. Afterwards it is possible by microscopy to follow the development of new capillaries growing from the edge of the cornea towards the angiogenesis-stimulating source. The cornea assay has the advantage of permitting non-invasive long-term monitoring of neoangiogenesis or tumor angiogenesis without interference of immunological interactions, as the cornea is an immunologically privileged site.

### 9.3. Mouse Hindlimb Ischemia Model

Ischemia stimulates angiogenesis and the mouse hindlimb inschemia model is a convenient model, which allows basic aspects of angiogenesis to be studied together with basic aspects of ischemia and reperfusion [100].

### 9.4. Matrigel Implantation Assay

Matrigel, which is liquid at 4 °C but solidifies to a gel at 37 °C, can be injected subcutaneously into animals together with tumor cells, angiogenic growth factors or angiogenesis inhibitors. Alternatively, the matrigel can be injected into special chambers and stored at room temperature until the matrigel solution has solidified and formed a gel-capsule that can subsequently be implanted s.c. in the animal. Although matrigel is not a typical site of pathological angiogenesis *in vivo*, it creates a suitable model for tumor angiogenesis as it replicates a potential hypoxic tumor microenvironment [97,98].

### 9.5. Animal Models of Tumor-Angiogenesis

Different animal models exist for studying angiogenesis [99,100,101]. In mice, it is possible to investigate the effect of angiogenesis not only on murine tumors but also on human tumors, if they are heterotransplanted to immunodeficient mice. Syngeneic models are most relevant for preclinical studies, but observed effects may be direct or indirect, including effects on the host immune system. An advantage of using human tumors heterotransplanted to immunodeficient mice is that it allows the investigation of tumor blood supply without interference of immune reactions between tumor and host.

We investigated whether levamisole could inhibit angiogenesis and tumor growth *in vivo* in nude mice heterotransplanted with a metastasizing human breast adenocarcinoma [75]. Subcutaneous administration of 12 mg/kg levamisole thrice a week for four successive weeks resulted in significantly inhibited tumor growth, whereas no significant reduction in tumor growth was observed in mice treated with 1.2 mg/kg levamisole or vehicle (PBS). Estimation of tumor vessel density revealed a significantly lower vessel density in tumors from mice treated with 1.2 mg/kg or 12 mg/kg levamisole than in tumors from untreated or PBS-treated mice. The use of immunodeficient mice as tumor hosts permitted the discrimination between a possible anti-angiogenic effect of levamisole and its assumed immune-stimulatory effect [102,103,104,105].

## 10. Clinical Use of Angiogenesis Inhibitors

The goal of much angiogenesis research is to identify clinically useful inhibitors and bring these into clinical trials [1]. Anti-angiogenic therapy is appealing for cancer treatment in combination with traditional chemotherapy, since tumor growth beyond the size of 1–2 mm in diameter (approximately 10^6^ cells) and metastasis depend on angiogenesis. A combination of traditional anti-cancer treatment and anti-angiogenic therapy targets both the proliferating cancer cells and the genetically stable ECs that are less prone to develop treatment-related resistance than tumor cells. Furthermore, treatment with a combination of angiogenesis inhibitors might be an advantage, since a tumor that initially produces a single angiogenic growth factor, e.g., VEGF, might over time mutate and start to produce other angiogenic growth factors as well [1].

Several angiogenesis inhibitors have shown clinical effects and many more are undergoing clinical evaluation [1]. The recombinant humanized VEGF antibody, bevacizumab (avastin) is approved as a first-line treatment for patients with colorectal cancer and lung cancer and is in clinical trial for several other cancer types. However, treatment with bevacizumab may cause serious adverse events related to coagulation/bleeding, wound healing, hypertension, proteinuria and hypersensitivity reactions [106,107].

Theoretically, it may be possible to identify more safe clinically effective small molecule angiogenesis inhibitors with equal or improved effectiveness compared to VEGF antibodies. The finding that levamisole inhibits EC tube formation by inducing a morphology identical to that induced by antibodies to VEGF makes levamisole interesting with respect to antiangiogenic activity *in vivo*. Levamisole was developed as an antihelminthic agent but has been used for adjuvant chemotherapy of colorectal cancer [73,74,75]. It has been replaced by other agents but with the knowledge of its anti-angiogenic action, it may be worth to reevaluate its potential for chemotherapy and/or search for more potent analogues.

## 11. Discussion

Angiogenesis is a complex process involving multiple pro- and anti-angiogenic factors. Since tumors are dependent on angiogenesis, inhibition of it is a valuable supplement to existing cancer treatment modalities.

Several assays exist for studying different aspects of angiogenesis and for identifying potential inhibitors. Animal models can be used for studying most aspects of angiogenesis and for testing both direct and indirect inhibitors. However, the limitations and expenses of such models should be taken into consideration. Using *in vitro* assays, many indirectly acting factors cannot be identified, but such assays allow easy investigation of various endogenous and exogenous inhibitors.

The coculture assay with HUVEC on a monolayer of NHDF has several advantages. It is easy and robust and it can distinguish between general apoptotic/cytotoxic effects and specific effects on ECs. The assay is quantitative and can be developed with BCIP/NBT to allow a semiquantitative evaluation of EC morphology by microscopy. The assay focuses on direct effects and distinguishes morphologically between differentiation and proliferation. A disadvantage of the assay is that CD31 does not distinguish between inhibition of differentiation and inhibition of proliferation (elongation). However, this is compensated by the possibility of microscopic evaluation. The use of markers distinguishing between clusters of ECs and differentiated ECs would further improve the assay.

Due to the use of a monolayer of confluent NHDFs, it appears that the coculture assay is insensitive to several indirectly acting pro-angiogenic factors, e.g., FGF, and it does not respond to indirectly acting inhibitors. Thus, several endogenous angiogenesis inhibitors, which may act by influencing ECM remodelling (angiostatin, antithrombin, endostatin, vasostatin), showed no effect in the assay.

Investigation of directly acting angiogenesis inhibitors in the coculture assay revealed that they fell in two groups according to their effect on EC morphology giving either clusters of non-differentiated cells or short non-canalized cords (Table 2, Figure 5).

**Table 2:** Morphological effect of angiogenesis inhibitors in the coculture assay.

Cluster morphology	Short cord morphology
Levamisole	NSAIDs
*N*-methyllevamisole	Platelet factor 4
p-Bromolevamisole	PTPI I, II
PTPI IV	Rapamycin
sVEGFR1	Suramin
SU5614	Thrombospondin
VEGF antibodies	TNP-470

**Figure 6:**
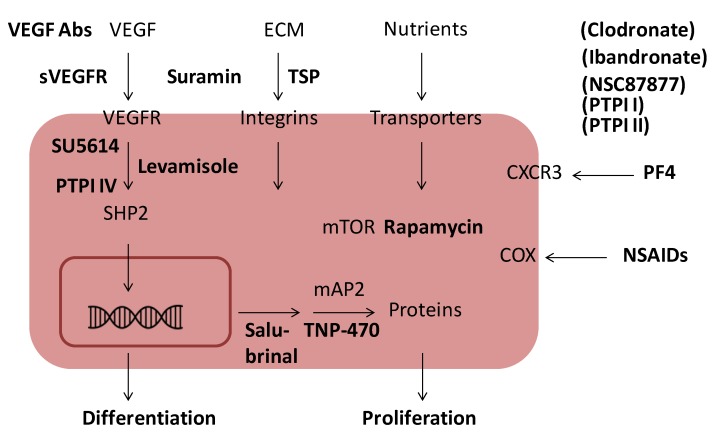
Schematic presentation of the influence of angiogenesis inhibitors on EC signaling. Important signaling pathways in ECs are the VEGF pathway, the ECM integrin pathway and the mTOR pathway. Blocking of the VEGF pathway by VEGF antibodies, SU5614, levamisole or PTPI IV inhibits differentiation. Blocking of ECM integrin signaling by TSP or mTOR signaling by rapamycin inhibits proliferation as does salubrinal and TNP-470. PF4, suramin, NSAIDs, clodronate, NSC87877, PTPI I, PTPI II inhibit EC proliferation in a morphologically similar way, while ibandronate gives mixed morphology, but the exact relationships between these with the major signaling pathways are unknown.

Treatment with antibodies to VEGF, sVEGFR, SU5614, PTPI IV or levamisole inhibited the formation of an EC capillary-like network and resulted in the formation of undifferentiated cell clusters of variable size. Therefore, these agents may block a common signalling pathway (*i.e.*, the VEGF pathway), presumably involving SHP-2, a target of PTPI IV and a known interaction partner of VEGFRs [10] (Figure 6). The anti-angiogenic mechanism of levamisole is unknown, but it most likely inhibits the VEGFR signalling pathway. The identification of the anti-angiogenic mechanism used by levamisole and its derivates will be an important topic of future experiments.

Treatment with PF4, rapamycin, suramin, TSP, TNP-470, PTPI I, PTPI II, chlodronate, salubrinal and the NSAIDs aspirin, ibuprofen and indometacin resulted in the formation of short non-canalized cords of differentiated ECs. These angiogenesis inhibitors have been shown to have different modes of action (Figure 6). Rapamycin inhibits mTOR-mediated EC proliferation [87]. TNP-470 covalently binds and inactivates methionine aminopeptidase 2 (MetAP-2) and thus prevents removal of the initiator methionine from as yet unidentified proteins involved in EC proliferation [65,66]. These events lead to a lack of EC proliferation and network formation as observed in the coculture assay. Similarly, NSAIDs by inhibiting COXs, inhibit EC proliferation. PTPI I and II and NSC87877 may target SHP1 (and other phosphatases), while salubrinal targets PP1/GADD34 [82].However, despite their different targets, these inhibitors appear to affect the same pathways leading to EC proliferation.

## 12. Conclusions

In conclusion: Angiogenesis inhibitors with an effect in the coculture assay could be divided in two groups according to morphology and a comparison of EC signalling pathways with known or suspected targets of these inhibitors indicated that the two groups consisted of inhibitors of VEGF signalling/differentiation and inhibitors of ECM/nutrients signalling/proliferation (Table 2, Figure 6). As a corollary, a primary action of VEGF in the coculture assay is to maintain differentiation of ECs and prevent reversion to an EPC like state. This may serve as a guideline for future research on small molecule angiogenesis inhibitors, which mimic the action of VEGF antibodies and have fewer side effects.
